# Tumor heterogeneity: next-generation sequencing enhances the view from the pathologist’s microscope

**DOI:** 10.1186/s13059-014-0463-6

**Published:** 2014-10-01

**Authors:** Samuel Aparicio, Elaine Mardis

**Affiliations:** Department of Molecular Oncology, BC Cancer Agency, 675 W10th Avenue, V5Z 1L3 Vancouver, British Columbia Canada; Nan and Lorraine Robertson Chair, Canada Research Chair, Department of Pathology and Laboratory Medicine, University of British Columbia, V6T 2B5 Vancouver, British Columbia Canada; The Genome Institute at Washington University, Washington University School of Medicine, 4444 Forest Park Blvd, St Louis, MO 63108 USA

The term heterogeneity covers many aspects of the variability in tumor phenotypes, which are a characteristic of human malignancies. Morphologists of the late 19^th^ century first described the multiple cell types composing tumors and began to recognize cancers of different types. Over the past half century the molecular underpinnings of the variability in human cancers has been gradually revealed but within the last 5 years there has been an explosion in our ability to determine and learn from cancer heterogeneity, through the use of next-generation sequencing and related methods. The complexity and variation in the structure of cancers can seem daunting, but important lessons in cancer biology and the approaches to therapy can be learned from studying how much of the complexity is subject to change and how much is a consequence of stochastic rather than deterministic processes. The evolution of clones, individual variation in response to therapy, distinct biological subtypes of cancer and tumor immune responses are all examples of the heterogeneous nature of human cancers. Self evidently, when heterogeneity is used to describe any aspect of a cancer, it is important to know which variational feature is being addressed.

## Experimental approaches to heterogeneity

A review by Hiley *et al.* [1] sets the stage for the research studies of heterogeneity published in this special issue, by updating the reader regarding how the use of next-generation sequencing and clever experimental design have increased our understanding of genomic and regional heterogeneity in cancers. The special collection provides several research-based studies of tumor heterogeneity, encompassing the variation between individuals (the tumor subtype) in breast cancers (Ali *et al.* [2]) and, by contrast, the lessons that can be learned from longitudinal study of single patient (‘N of 1’) cases (Fisher *et al.* [3], Nadauld *et al.* [4]). These studies provide contrasts between the approaches needed to determine disease groupings in populations, where many hundreds or thousands of patients must be studied, with the approaches to pursuing the moving target of individual cancers. The latter can be effectively studied in smaller numbers with informative consequences when evolution is used to sift the features undergoing selection and fixation. An Opinion piece by Good *et al.* [5] provides a scientific and philosophical perspective on the N of 1 paradigm.

## Methodological approaches to heterogeneity

Of equal importance to the experimental approaches used to study heterogeneity are the methods used to evaluate heterogeneity across the spectrum of variation that can be measured in cancer by different assay types. These encompass next-generation sequencing methods for analyzing tumor and normal cell composition, the analysis of clonal populations in cancers (Qiao *et al*. [6]), and methods addressing epigenetic plasticity (Zheng *et al*. [7]). Hence, several important tools and approaches are presented that facilitate answering critical questions about heterogeneity, and an opinion piece contributed by Russnes *et al.* [8] advocates for data integration to better interpret heterogeneity data.

## Single cell approaches to heterogeneity

Resolving structure and function with single cell approaches is also becoming important, both in studies of clonality as well as for functional assessment of tumor cell populations. Learning how to reconcile whole tumor cell-based population approaches with the single cell data will be important. Nicholas Navin’s review [9] of single cell sequencing in cancer studies provides a survey of this rapidly developing area.

## RNA-based studies of heterogeneity

Next-generation sequencing-based studies of RNA populations from cancer cells are revealing important aspects of transcriptional activity and its role in cancer. A review by Patrick Nana-Sinkam and Carlo Croce [10] sets the stage by discussing the role of microRNAs in gene regulation in cancers. White *et al.* [11] present important new descriptions of long non-coding RNAs (lncRNAs) in lung cancers, and Wyatt *et al.* [12] describe transcriptomes in the context of therapy response in high-risk prostate cancers. The method for detecting allele-specific expression contributed by Mayba *et al.* [13] will also yield important insights into which variants detected by DNA sequencing are actually being expressed in the transcriptome of cancer cells. The role of the epigenome in contributing to the patterning of transcriptomes and the possibility of modulating RNA expression is emphasized in three primary research articles exploring this aspect of tumor heterogeneity (Lund *et al.* [14] , Fleischer *et al.* [15], and Charlton *et al.* [16]).

## Clinical aspects of heterogeneity

As our underlying knowledge about cancer genomics and heterogeneity improves, the need to translate this information into informed cancer care for patients is an obvious next step. Berger and Varghese have contributed an Opinion piece [17] to describe the translation of cancer genomics in the clinic, and contributions from de Bono [18] and Bardelli [19] outline the use of circulating tumour cells (CTCs) and circulating free DNA (cfDNA), respectively, as approaches to monitoring tumor progression. These blood-based or ‘liquid biopsy’ approaches present an exciting new paradigm in contrast to conventional and less sensitive imaging-based approaches to monitor patients. Deininger also reviews [20] an important area to clinical therapeutics that is often identified by genomic information, providing an overview of therapy response and resistance to targeted therapies. The genomic variability between patients is highlighted in the research article of Ali *et al*. [2], delineating molecular subtypes in large cohorts of breast cancer patients.

## Data and more data

Finally, in the genomic era, sharing of data and complete descriptions of analytic methods, to the level of providing code in addition to data, will prove crucial to continued success. Boutros *et al.* [21] provide a novel look at crowd-sourcing algorithms for cancer analysis, and Bartha Knoppers and colleagues [22] present a critically important Opinion regarding the legal framework for genomic data sharing. Hence, the special collection provides a wide-ranging overview of cancer heterogeneity in its many manifestations, from fundamental methods-based approaches to study heterogeneity to the use of genomic information for response and progression monitoring. We think the breadth of cancer studies that have been impacted by next generation sequencing, including improved understanding and characterization of cancer heterogeneity, is setting the stage for major breakthroughs in our biological understanding of this vexing and complicated disease.
